# The effect of Zentangle on cognitive focus, emotional well‐being, and stress levels: A neural perspective

**DOI:** 10.1002/brb3.3628

**Published:** 2024-08-21

**Authors:** Muhammad Usman, Tzzy‐Ping Jung, Ding‐Yun Hsin, Chun‐Ling Lin

**Affiliations:** ^1^ International Ph.D. Program in Innovative Technology of Biomedical Engineering and Medical Devices Ming Chi University of Technology New Taipei City Taiwan; ^2^ Institute for Neural Computation and Institute of Engineering in Medicine University of California San Diego San Diego California USA; ^3^ Taipei Municipal Yangming High School Taipei City Taiwan; ^4^ Department of Electrical Engineering Ming Chi University of Technology New Taipei City Taiwan; ^5^ Department of Electronic Engineering National Taipei University of Technology Taipei Taiwan

**Keywords:** art therapy, electroencephalography, mediation, mindfulness, well‐being, Zentangle

## Abstract

**Purpose:**

This study aimed to assess how a Zentangle intervention influences cognitive focus, emotional well‐being, stress levels, and neural activity patterns across brain regions and frequency bands.

**Method:**

A cohort of 30 healthy adults, all without prior Zentangle experience, participated in this study. Electroencephalography (EEG) was used to measure their brain activity, and self‐reported data were collected through questionnaires to assess subjects’ concentration levels, emotional calm, and stress and anxiety.

**Findings:**

Participants reported enhanced cognitive focus and emotional well‐being, evidenced by increased self‐reported concentration and emotional calmness, and reduced stress and anxiety levels during the intervention. EEG analyses revealed notable changes in neural activity patterns, including decreased delta power and increased theta, alpha, beta, and gamma bands. Functional connectivity analysis also highlighted alterations in the brain's functional connectivity, suggesting potential effects on neural communication and information processing.

**Conclusion:**

This study provides compelling evidence of Zentangle's impact on EEG data, aligning it with equanimity and tranquility consistent with previous mindfulness research. These findings underscore Zentangle as an effective mindfulness practice, potentially enhancing cognitive focus and emotional well‐being, and emerging as a valuable intervention for improving mental health and overall well‐being.

## Graphical abstract

Zentangle, a mindfulness‐based art therapy, positively impacts cognitive focus, emotional well‐being, and brain activity in adults. EEG data reveal increased theta, alpha, beta, and gamma bands and altered functional connectivity, aligning Zentangle with equanimity and tranquility, offering potential mental health benefits.

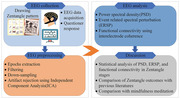

## 1 INTRODUCTION

### 1.1 Zentangle: a fusion of mindfulness meditation and art therapy

Zentangle is a creative process incorporating mindfulness meditation and art therapy, introduced by Rick Roberts and Maria Thomas in 2003. This artistic practice involves the creation of intricate patterns on small paper tiles, drawing a Zentangle as a meditative and calming creative process that combines focused awareness with artistic expression (Roberts & Thomas, 2012). The amalgamation of Zen's essence, represented by rhythmic breath and the intricacy of tangle patterns, encapsulates a creative endeavor that resonates with Hall's perspective, highlighting an internal connection and tranquility (Hall, 2012; Hesterman & McAuliffe, 2017). The synergy between principles of art therapy and mindfulness meditation, as applied to Zentangle, is an evolving research domain with promising potential for addressing various mental health conditions (Kopeschny, 2016). The foundation of this integration is rooted in the theoretical framework advanced by Peterson and Davis, which delved into the philosophical, neuropsychological, and physiological dimensions of mindfulness, coupled with art's capacity to impact brain plasticity and address trauma (Davis, 2015; Peterson & Rappaport, 2014).

A search for “Zentangle” across multiple search engines yielded only six relevant articles, demonstrating the need to explore this intriguing practice further. In an initial study, engagement in Pastel Nagomi art and Zentangle sessions was associated with enhanced positive affect and reduced negative affect and depression among undergraduates, despite acknowledging potential session distribution bias (Cheung et al., 2023). Another investigation showed that Zentangle art effectively mitigated stress, bolstered self‐efficacy, and improved well‐being among healthcare workers, underscoring its cost‐effective and advantageous role as an intervention (Hsu et al., 2021). Additionally, a separate inquiry focused on the successful transition of Zentangle workshops from in‐person to online formats, highlighting favorable participant experiences and satisfaction with integrating information and communication technology (Sit et al., 2022). Furthermore, the foundational Zentangle method exhibited efficacy in diminishing depressive symptoms and fostering self‐compassion in older adults, suggesting its potential as an alternative therapeutic avenue for mental health (Chan & Lo, 2024). An additional pilot study found that an 8‐week Zentangle program reduced psychiatric symptoms and increased mindfulness in people with serious mental illness (Stojcevski et al., 2023). Finally, a comprehensive exploration showed Zentangle's capacity to enhance affective well‐being by reducing negative affect and anxiety while concurrently fostering self‐compassion (Chung et al., 2022). Overall, Zentangle holds promise as a therapeutic practice with wide‐ranging mental health benefits. It warrants further research in this area to better understand its potential applications and mechanisms of action.

### 1.2 Mindfulness and mental behavior

The growing popularity of alternative and complementary treatments for managing mental health conditions has given rise to mindfulness meditation, a deliberate practice that directs nonjudgmental attention to the present moment, nurturing innate awareness (Kabat‐Zinn & Hanh, 2009). Mindfulness meditation has shown its effectiveness in several areas, including stress reduction (Bohlmeijer et al., 2010; Irving et al., 2009; Schell et al., 2019), the promotion of emotional serenity (Hill & Updegraff, 2012; Luberto et al., 2021), and the management of depression (Desrosiers et al., 2013; Thompson et al., 2015) and anxiety (Brown & Ryan, 2003; Cotton et al., 2020) in individuals with adverse mental and physical health challenges. Furthermore, research has shown that individuals characterized by both high‐trait mindfulness and low‐trait anxiety exhibit improved cognitive performance. This improvement encompasses various aspects, including conflict control and working memory capacity (Jaiswal et al., 2018; Jha et al., 2010; Mrazek et al., 2013).

### 1.3 Electroencephalography (EEG) and mindfulness mediation

Electroencephalography (EEG) is a noninvasive technique that measures the brain's electrical activity through electrodes placed on the scalp (Teplan, 2002). In mindfulness meditation, EEG is used to observe variations in brainwave patterns, which reflect different mental, conscious, physiological, and pathological states (Dunn et al., 1999; Lomas et al., 2015; Travis & Shear, 2010; Zaccaro et al., 2018). Many studies in mindfulness research consistently exhibit a noteworthy decrease in delta band activity alongside increases in theta, alpha, beta, and gamma band activity. For instance, mindfulness‐based stress reduction (MBSR) training increases alpha and beta EEG power while reducing delta power, suggesting enhanced coordination between brain and heart activities (Gao et al., 2016). MBSR is also associated with elevated resting‐state beta powers and reduced delta power (Ng et al., 2021). High levels of mindfulness correlate with reduced anxiety, decreased delta activity, and augmented alpha activity, implying heightened attentiveness, improved conflict control, and enhanced working memory capacity (Jaiswal et al., 2019).

Combining EEG with respiration signals enhances discrimination between meditation and control conditions, potentially reflecting meditation proficiency, with spectral analysis underscoring increased beta and theta EEG power during meditation (Ahani et al., 2014). Neuroelectric and imaging research highlights meditation's ability to slow overall brain activity and elevate theta and alpha activation, showing improved practice proficiency (Cahn & Polich, 2006). Systematic reviews have shown increased theta power in the frontal, parietal, and temporal lobes during mindfulness meditation (Chiesa & Serretti, 2010), with a correlation between heightened theta power and activity in the anterior cingulate cortex (Lomas et al., 2015). Additionally, mindfulness meditation has been linked to elevated theta power in the frontal and parietal lobes and experienced meditators exhibit reduced mind‐wandering alongside heightened theta power in these regions (Brandmeyer & Delorme, 2018; Ivanovski & Malhi, 2007). Moreover, certain techniques, such as pulsed‐wave chromotherapy and guided relaxation, have been associated with increased theta‐alpha oscillations during arrest reactions (Cheron et al., 2022). Furthermore, long‐term meditators have shown increased gamma power in the parietal‐occipital region during non‐rapid eye movement sleep, suggesting potential enhancement in attention, learning, and memory because of meditation (Ferrarelli et al., 2013).

Functional connectivity quantifies statistical dependencies between neuronal signals, whereas effective connectivity measures directed influence between neuronal systems (Schoffelen & Gross, 2009). There is a notable distinction between time‐ and frequency‐domain connectivity measures, with coherence being a prominent metric for quantifying oscillatory interdependencies among brain regions (Gross et al., 2001; Hoechstetter et al., 2004). Mindfulness meditation often leads to reduced functional connectivity between brain regions, as evidenced by diminished lagged coherence across different traditions and frequency bands. This suggests detachment and dissolution of ego boundaries during meditation (Lehmann et al., 2012). Breath‐counting mindfulness reduces intracortical lagged coherence but increases head‐surface coherence, signifying reduced cognitive‐sensory connectivity and heightened bodily attention, distinct from experienced meditators (Milz et al., 2014).

Zentangle is a mindfulness intervention informed by neurophysiology, blending mindfulness with creative expression through intricate pattern creation (Stojcevski et al., 2023). This practice offers a meditative experience that enhances focus and relaxation, making it particularly valuable for individuals struggling with traditional mindfulness. In this study, we aim to assess the feasibility of Zentangle as a potential mindfulness intervention and its effects on participants’ mental well‐being. To achieve this, we recruited 30 participants and divided their participation into three phases: early Zentangle (EZ), mid Zentangle (MZ), and late Zentangle (LZ). Our assessment of mental health includes various methods, such as self‐report questionnaires and EEG data analysis. Additionally, we aim to explore the suitability of Zentangle as an alternative approach for individuals who may find traditional mindfulness challenging. This study seeks to contribute to understanding Zentangle as a mindfulness practice and its potential benefits for mental well‐being. Furthermore, this research may have broader implications for mindfulness interventions and their impact on mental health.

## 2 METHODS

### 2.1 Participants

This study recruited 30 male participants with an average age of 24 ± 2 years from Ming Chi University of Technology, New Taipei City, Taiwan. All participants had normal or corrected‐to‐normal vision and exhibited a dominant right‐hand preference. They were required to be in good health with no history of gastrointestinal, cardiovascular, neurological, or psychological disorders. We verified that none of the participants had exposure to Zentangle before their involvement in the study. We thoroughly explained the experimental procedure and EEG data acquisition process during the Zentangle activity to ensure participants fully understood the study. It is important to note that we did not disclose the specific hypotheses under investigation to the participants. The research protocol with the identifier 202207EM003, implemented in this study, received approval from the Institutional Review Board of the Research Ethics Committee at National Taiwan University. Before commencing any experiments, we obtained informed consent from each participant.

### 2.2 Zentangle experiment

**FIGURE 1:**
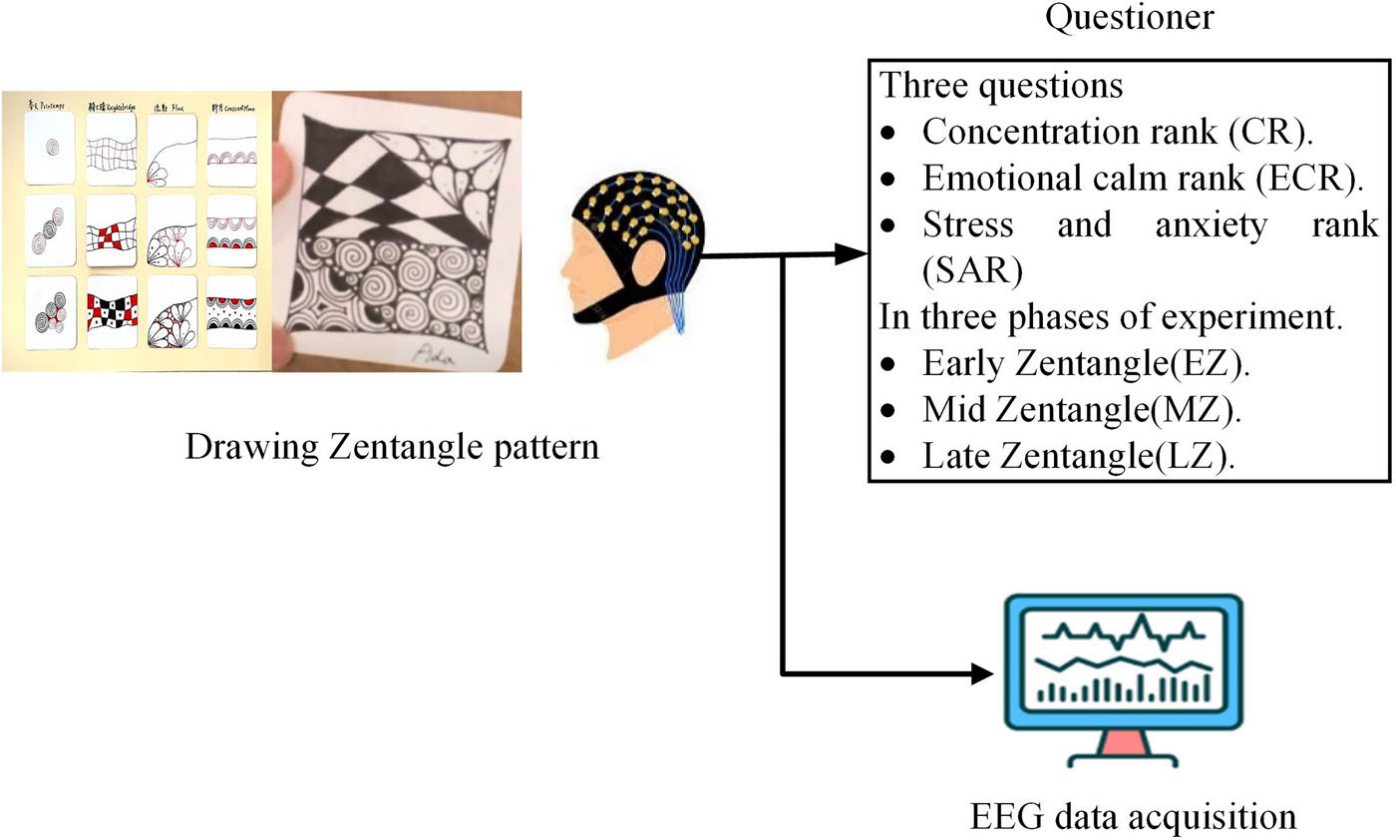
Designed Zentangle experiment in this study.

During the Zentangle experiment, participants sat comfortably in chairs before a 27″ computer monitor. As none of the participants had prior exposure to Zentangle, we provided a 20‐min instructional video to guide them in creating Zentangle patterns, available at this link: https://www.youtube.com/watch?v=ablB‐tkoS8U. Figure 1 illustrates the experimental design and four basic Zentangle patterns used in this study. Following the instructional video, the participants began creating their Zentangle artwork using 8.9 × 8.9 cm^2^ white paper. They had approximately 15–20 min for this task, and a reminder paper was on the table for reference. Upon completing their Zentangle creations, participants rated their concentration, emotional calmness, and stress reduction during the experiment. We gathered EEG data to examine participants’ brain activity, whereas they engaged in the creative process.

### 2.3 Questionnaire

In this study, we assessed the experiment's performance using three questions:

1. The first question assessed the level of concentration rank (CR).
2. The second question examined the level of emotional calm rank (ECR).
3. The third question focused on stress and anxiety rank (SAR).

We asked participants to rate their levels of CR, ECR, and SAR on a 10‐point Likert scale, where 1 represents the lowest score and 10 represents the highest score, both before and during the experiment. A table summarizing the self‐reported questionnaire is presented in the Table A. This subjective self‐assessment aimed to provide insights into the impact of Zentangle on these psychological factors. Although we acknowledge that more objective physiological measures could offer additional insights, the chosen method allows for a straightforward and accessible evaluation of participants’ perceived changes in concentration, emotional calm, and stress levels throughout the experiment. Future research on this topic will incorporate physiological measures to complement and enhance subjective assessment, furthering our understanding.

### 2.4 EEG data acquisition and preprocessing

Throughout the Zentangle experiment, we continuously recorded 15‐min EEG signals from each participant using a NeuroScan system (NuAmps Compumedics Ltd.). The system featured 32 active electrodes positioned on the headcaps according to the extended 10–20 system. We sampled EEG signals at a rate of 1 kHz and digitized them at a 32‐bit quantization level. We applied a notch filter set at 60 Hz to reduce noise interference. We ensured that the contact impedance between the scalp and the electrode remained below 5 kΩ by using conductive gel.

**FIGURE 2:**
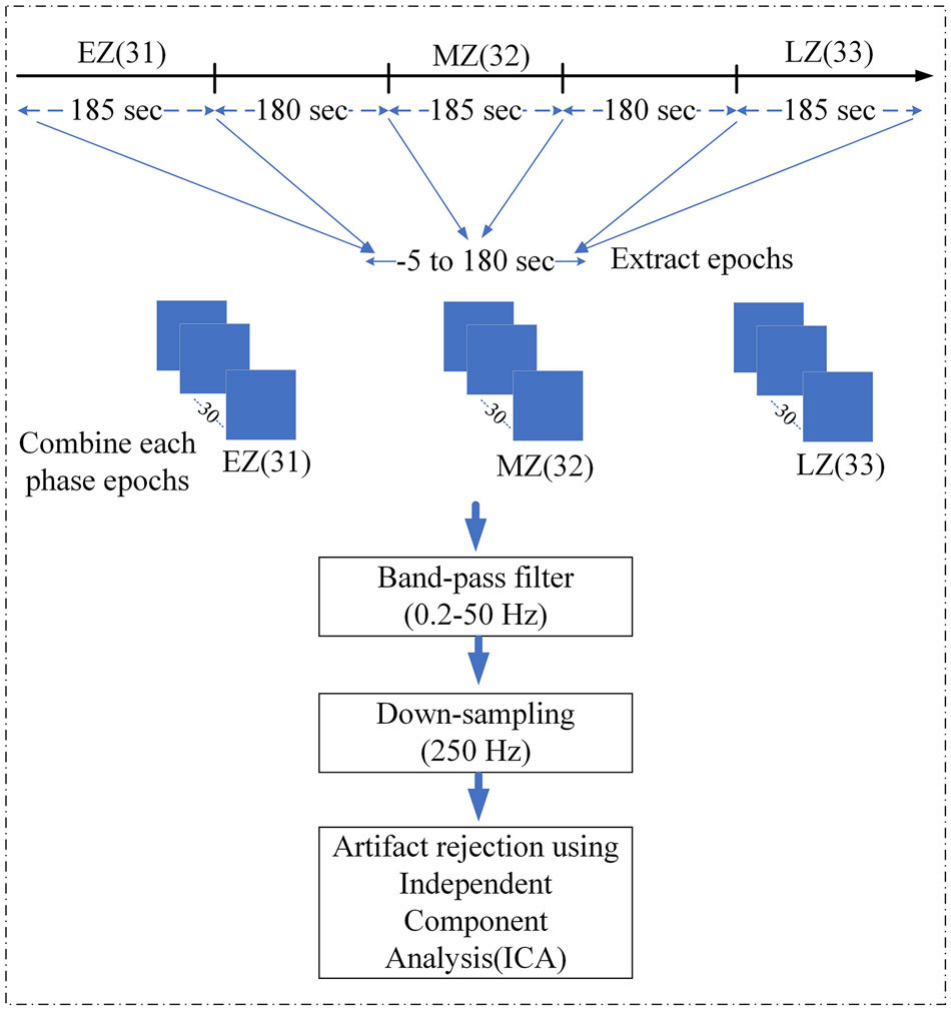
Flowchart of electroencephalography (EEG) preprocessing using EEGLAB and MATLAB to process data from events 31, 32, and 33 through the extraction of epochs, band‐pass filtering, down‐sampling, and Independent Component Analysis (ICA) artifact removal.

In this study, we preprocessed and analyzed the EEG data using EEGLAB, a tool provided by the Swartz Centre for Computational Neuroscience of the University of California San Diego (Delorme & Makeig, 2004), running within MATLAB. Figure 2 illustrates the flowchart of the EEG preprocessing in this study. The 15‐min EEG recording was segmented into three sections based on strategically placed event markers numbered 31, 32, and 33. These markers directly correspond to specific phases within the Zentangle activity:

1. EZ: This 185‐s segment (marked by event 31) reflects brain activity during participants’ initial engagement with the Zentangle task.
2. MZ: Following a 180‐s exclusion period after EZ, event marker 32 demarcates the beginning of the MZ phase. This 185‐s segment captures brain activity midway through the Zentangle experience.
3. LZ: The final 185‐s segment, marked by event marker 33 and following another 180‐s exclusion period, reflects brain activity towards the end of the Zentangle task.

Based on these events, we extracted epochs in each participant's data, each spanning −5 to 180 s in duration. Subsequently, we grouped the epoched data into three categories. The segmented data (epochs) underwent a band‐pass filter ranging from 0.2 to 50 Hz and down‐sampled to 250 Hz. To effectively remove artifacts, including channel noise, eye blinks, heart noise, and muscle noise, we used Independent Component Analysis and the independent component labeling tool, a plugin within EEGLAB. We reduced the influence of non‐brain sources by applying zero projection weights to each scalp channel. We then recalculated the initial data using a weighted linear combination of all 32 source electrodes. In our EEG data analysis, we used EZ as the baseline to observe and analyze the effects of Zentangle during conditions MZ and LZ.

### 2.5 EEG data analysis

To analyze the EEG data, this study calculated the power spectral density (PSD) of segmented data (epoch) using the *spectopo()* function implemented in EEGLAB. This function uses the MATLAB *pwelch()* function with a Hamming window. The data had a sampling frequency of 250 Hz, and each analysis window contained 250 samples, equivalent to a duration of 1 s. This study computed the PSD for specific EEG channels, including frontal (F3, Fz, F4), central (C3, Cz, C4), parietal (P3, Pz, P4), and occipital (O1, Oz, O2). Additionally, this study calculated the average PSD within specific frequency ranges: 1–4 Hz (delta), 4–8 Hz (theta), 8–13 Hz (alpha), 13–30 Hz (beta), and 30–50 (gamma).

This study used the *newtimf()* function in EEGLAB for time‐frequency analysis. The analysis spanned frequency ranges from 1 to 50 Hz and used the Morlet wavelet transform with 3 cycles at the lowest frequency and 30 cycles at the highest frequency. This approach generated 200 time points from −5 to 180 s and estimated 200 log‐spaced frequencies ranging from 1.0 to 50.0 Hz. This study selected event‐related spectral perturbation (ERSP) and time‐frequency data (tfdata) for time points where time (*t*) was greater than or equal to 0 s. Subsequently, ERSP and tfdata within specific frequency ranges (1–4, 4–8, 8–13, 13–30, and 30–50 Hz) were extracted for further analysis.

In this study, we extensively investigated functional connectivity using coherence analysis. This analysis aimed to evaluate synchronization patterns among pairs of EEG channels within designated frequency bands. We quantified coherence using the Pearson correlation coefficient (*R*), a fundamental measure for assessing linear relationships (Sedgwick, 2012). MATLAB's *corrcoef()* function facilitated the computation of this correlation coefficient, which enabled a meticulous assessment of coherence between processed signals. We collected EEG channel signals using a band‐pass infinite impulse response Butterworth filter with a filter order of 50 and corner frequencies aligned with the specified frequency band. The formula used to calculate coherence between channel *X* and *Y*, denoted as *R(X, Y)*, succinctly captures the essence of the Pearson correlation coefficient:

$$R\left(X,Y\right)=\frac{1}{N-1}\sum_{i=1}^{N}\left(\frac{Xi-mX}{sX}\right)\left(\frac{Yi-mY}{sY}\right)$$

where *mX* and *sX* are the mean and standard deviation of *X*, respectively, and *mY* and *sY* are the mean and standard deviation of *Y*. N is the total number of data points, representing the number of pairs (X,Y) in the data set. The index i ranges from 1 to N, indicating each individual data point in the data set. The value of *R* ranges from 0 to 1, representing no coherence to perfect coherence, respectively.

### 2.6 Statistical analysis

To assess the suitability of the parameters, including questionnaire data and EEG data, for parametric or nonparametric analyses, Kolmogorov–Smirnov and Shapiro–Wilk tests were performed to examine the normality of the data (Massey, 1951; Razali & Wah, 2011). The results showed high significance (*p *< 0.05), suggesting that the data were non‐normally distributed. For the questionnaire data (CR, ECR, and SAR levels), we used the Wilcoxon sign rank test to examine whether there were differences between the three phases (EZ, MZ, and LZ) of the Zentangle experiment (Woolson, 2005). This nonparametric test was appropriate for our non‐normally distributed data.

In analyzing PSD and ERSP data, this study also employed the Wilcoxon sign rank test to assess whether there were statistical differences between the three phases of the Zentangle experiment. In functional connectivity analysis, this study applied two statistical thresholds. The first threshold selected channel pairs with coherence values greater than 0.7. The second threshold involved using the Wilcoxon sign rank test to assess the coherence values of these selected channel pairs across the three phases of the Zentangle experiment. A functional connectivity analysis included channel pairs with *p* < 0.05 as statistically significant.

## 3 RESULTS

### 3.1 Analysis of questionnaire

**FIGURE 3:**
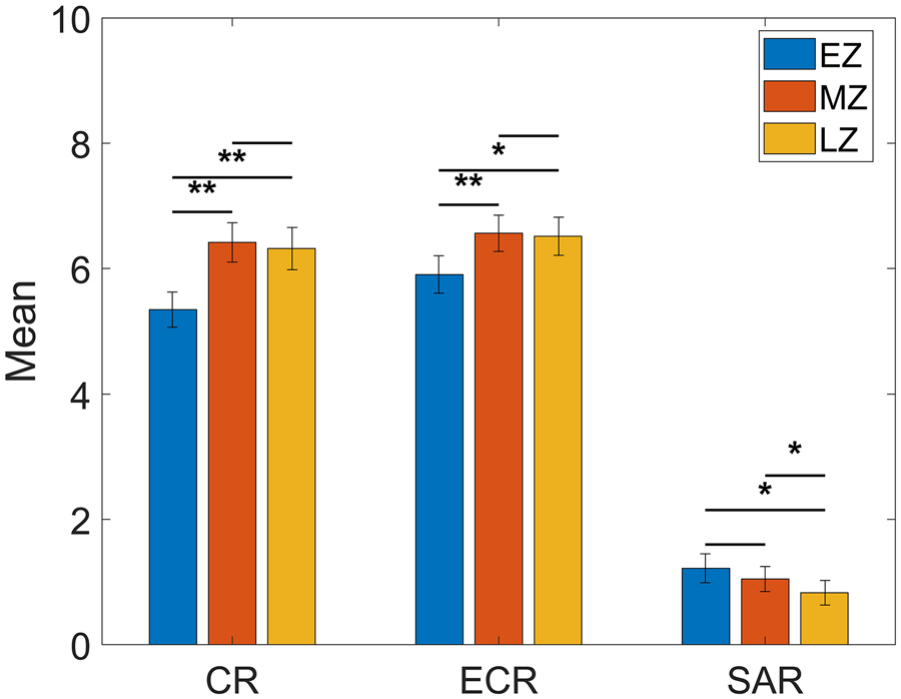
Mean values of participants’ concentration rank (CR), emotional calm rank (ECR), and stress and anxiety rank (SAR) levels during three sections of the Zentangle experiment: early Zentangle (EZ), mid Zentangle (MZ), and late Zentangle (LZ). Significance differences are denoted by (**) for *p* < 0.01 and (*) for *p* < 0.05. Standard deviation is also calculated.

Figure 3 shows statistically significant differences in participants’ CR, ECR, and SAR according to the questionnaire results. Specifically, significant improvements in CR were observed between the EZ and MZ sections (*p *< 0.01) and between the EZ and LZ sections (*p *< 0.01). Regarding ECR, we observed a significant difference between the EZ and MZ sections (*p *< 0.01) and between the EZ and LZ sections (*p *< 0.05). SAR displayed noteworthy reductions in stress and anxiety between the EZ and LZ sections (*p *< 0.05) and between the MZ and LZ sections (*p *< 0.05), with no significant difference detected between the EZ and MZ sections.

### 3.2 Power spectral density analysis

**FIGURE 4:**
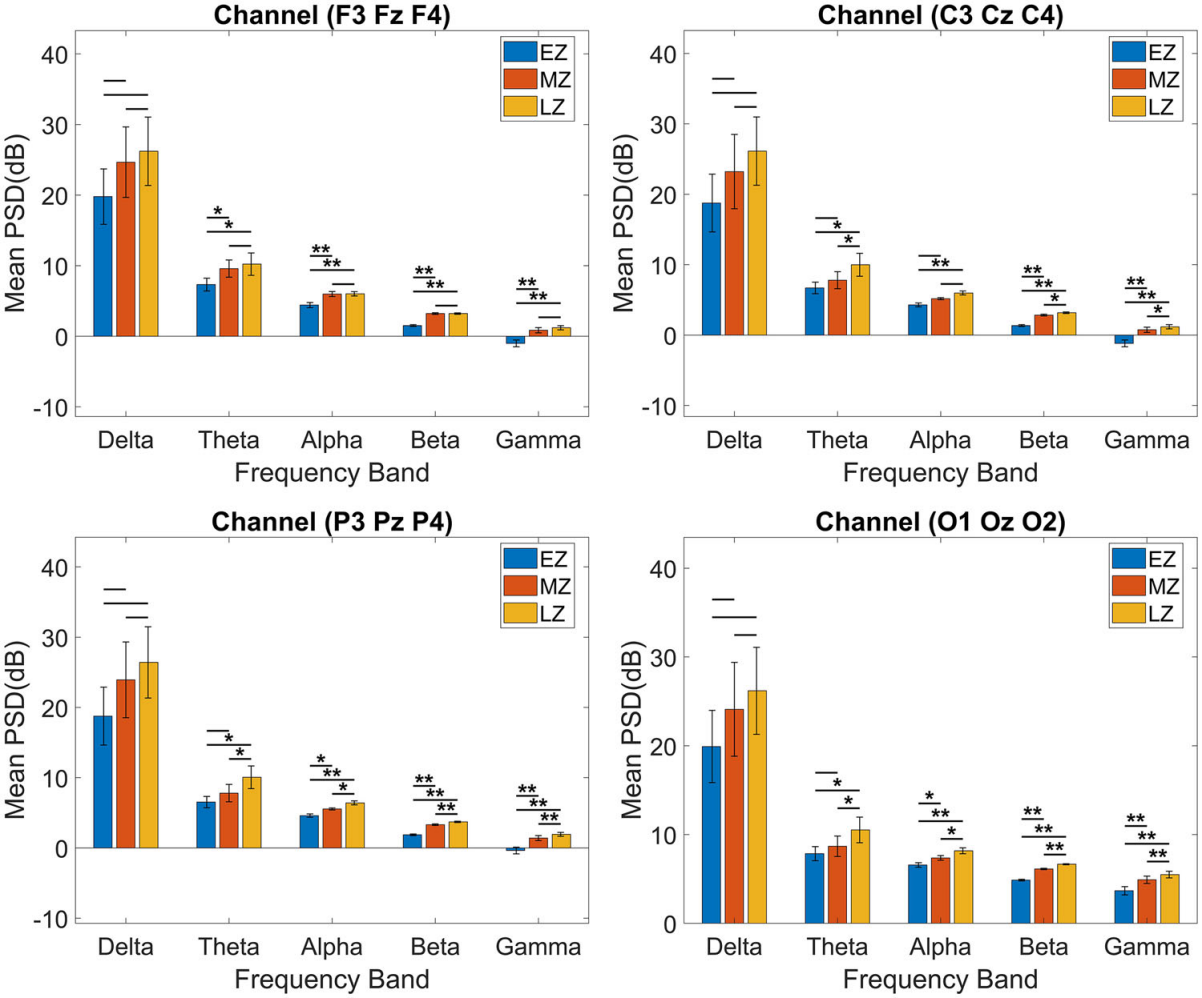
Mean power spectral density (PSD) values across different brain regions and frequency bands, with standard deviation, are calculated. Statistical significance of differences among early Zentangle (EZ), mid Zentangle (MZ), and late Zentangle (LZ) in each frequency band is denoted by (**) for *p* < 0.01 and (*) for *p* < 0.05.

Figure 4 shows the PSD patterns recorded from electrodes placed over specific brain regions, including the frontal (F3, Fz, F4), central (C3, Cz, C4), parietal (P3, Pz, P4), and occipital (O1, Oz, O2) loops. We meticulously computed the mean PSD values for each region and conducted a thorough comparative analysis across five frequency bands, focusing on the three segments: EZ, MZ, and LZ. Notable variations in mean PSD values emerged within each loop. In the frontal loop, the theta band exhibited significant differences between EZ versus MZ and EZ versus LZ (*p *< 0.05). Similarly, we noted the same trend between EZ versus LZ and EZ versus MZ (*p <* 0.01) for the alpha, beta, and gamma bands. As for the central loop, there were significant differences in theta band between sections EZ and LZ and MZ and LZ (*p *< 0.05). EZ and MZ showed noteworthy distinctions in the alpha band (*p *< 0.01). The beta and gamma bands also showed considerable dissimilarities between sections EZ versus MZ, EZ versus LZ, and MZ versus LZ (*p *< 0.05, *p *< 0.01). Similarly, the parietal and occipital loops exhibited significant differences in the theta band, comparing sections EZ versus LZ and MZ versus LZ (*p *< 0.05). Similarly, the alpha band displayed remarkable variations between sections EZ versus MZ, sections EZ versus LZ, and sections MZ versus LZ (*p *< 0.05, *p *< 0.01). Additionally, substantial disparities in the beta and gamma bands were observed between sections EZ versus MZ, sections EZ versus LZ, and sections MZ versus LZ (*p <* 0.05). However, the delta band across all loops showed no significant differences.

### 3.3 Topographic map of ERSP analysis

**FIGURE 5:**
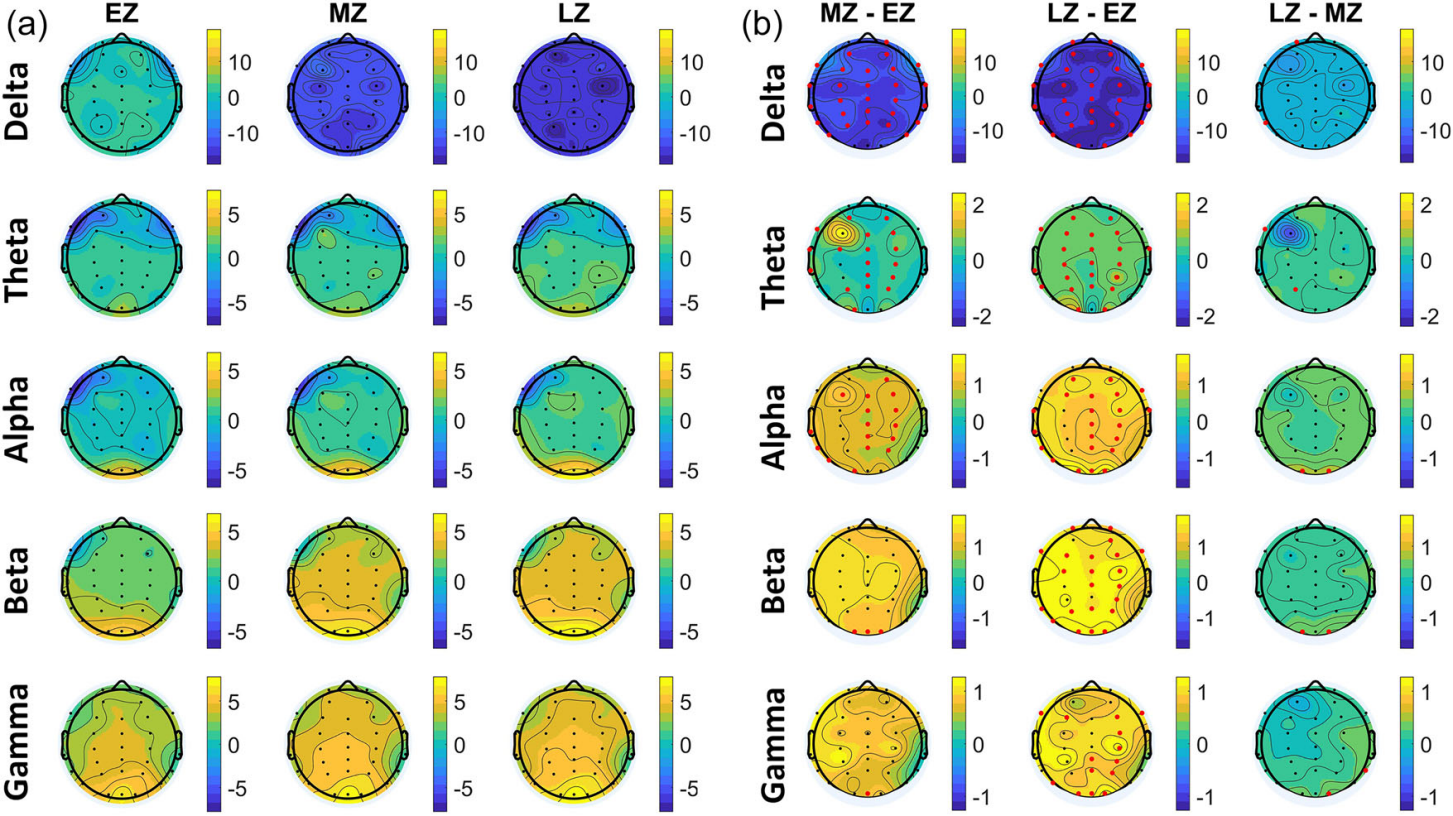
Event‐related spectral perturbation (ERSP) analysis of electroencephalography (EEG) data across different brain regions and frequency bands. Part (a) shows the difference in ERSP power among three sections (early Zentangle [EZ], mid Zentangle [MZ], and late Zentangle [LZ]) for each frequency band. Part (b) shows the topographical plots of the difference in ERSP.

Figure 5a presents the topographic map results of the ERSP analysis conducted on three distinct sections (EZ, MZ, and LZ) across five frequency bands. To visualize the difference in ERSP power among the three sections, Figure 5b illustrates the computed ERSP power differences for each frequency band. In the topoplots, significant disparities (*p < *.05) are indicated in red, whereas yellow represents maximum positive power, blue indicates maximum negative power, and green denotes power near zero.

The findings reveal several notable patterns:

- A marked reduction in delta power was observed for both section MZ and section LZ compared to section EZ, encompassing the entire scalp.
- A noteworthy elevation in theta power was observed across all electrodes in section MZ compared to section EZ (excluding prefrontal, right temporal, and occipital regions), as well as in section LZ compared to section EZ (excluding prefrontal and right temporal regions).
- In the alpha frequency band, a significant increase in power was observed in the frontal, central, right central, parietal, left temporal, and occipital regions for both sections MZ versus EZ and LZ versus EZ.
- In the beta frequency band, significant power increases were observed between MZ and EZ in the occipital region, between LZ and EZ in the frontal, central, right central, parietal, left temporal, and occipital regions, and between LZ and MZ in the occipital region.
- In the gamma band, section MZ versus EZ showed a significant increase in power solely in the central occipital channel. In contrast, section LZ versus MZ displayed a significant power increment in the left and right frontal, right central, right parietal, and occipital regions.

### 3.4 Functional connectivity analysis

**FIGURE 6:**
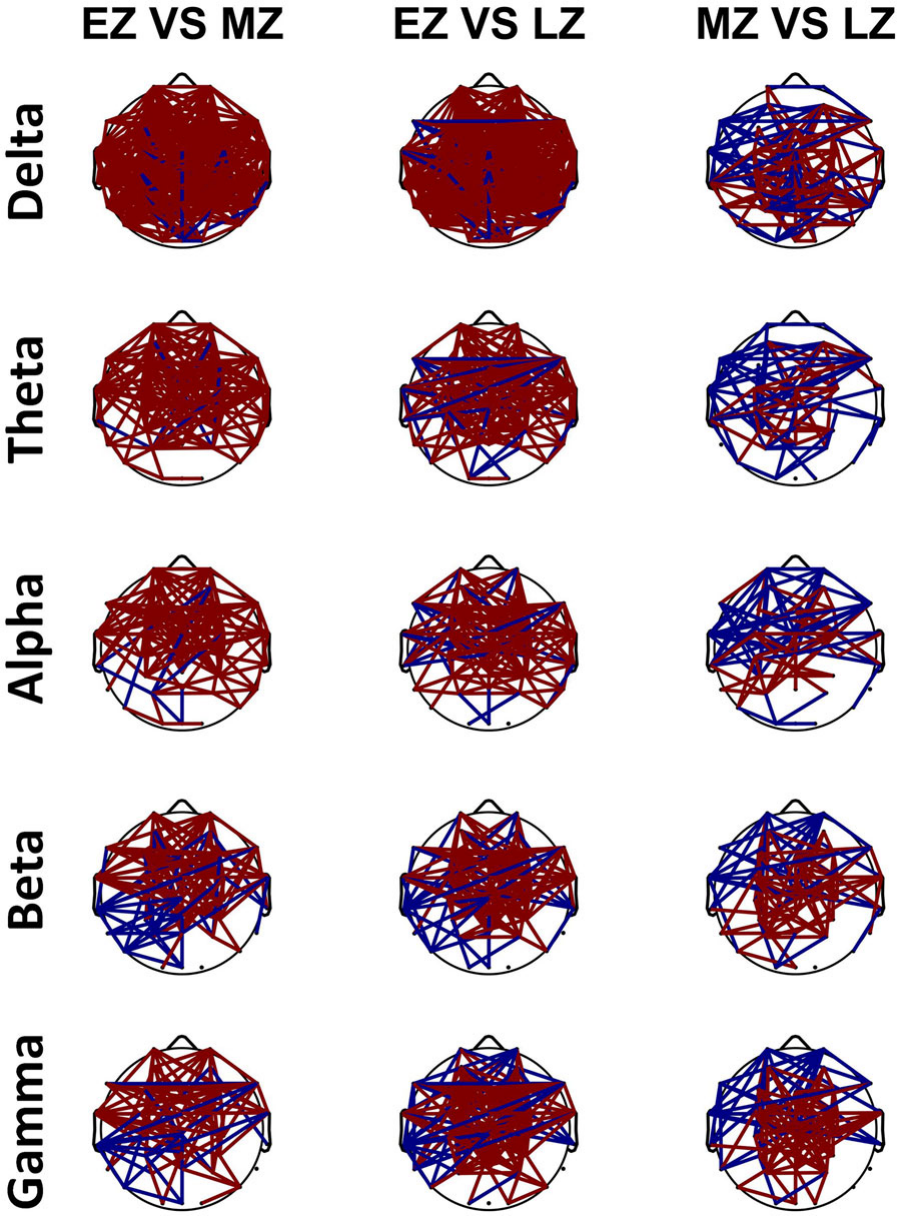
Functional connectivity among three distinct sections (early Zentangle [EZ], mid Zentangle [MZ], and late Zentangle [LZ]) in different frequency bands. The threshold value of coherence is *r* > 0.7. Red indicates stronger interelectrode coherence in the pre‐section compared to the post‐section, whereas blue represents the reverse situation.

This study conducted a comparative analysis of functional connectivity based on interelectrode coherence using EEG data from three distinct sections (EZ, MZ, and LZ). Figure 6 illustrates the functional connectivity computed within specific frequency bands (delta, theta, alpha, beta, and gamma) among three sections. We carefully selected all interelectrode coherence values to be greater than 0.7 (*r >* 0.7). The color scheme employed in Figure 6 assigns red to show stronger interelectrode coherence in the pre‐section compared to the post‐section, whereas blue represents the reverse situation. The analysis of between‐group interelectrode coherence revealed several key findings:

- Section EZ exhibited more synchronized oscillations with more functional connections across all frequency bands, in contrast to sections MZ and LZ.
- When comparing section EZ with section MZ, functional connectivity in the pre‐section was more pronounced in the lower frequencies (delta, theta, and alpha) compared to the higher frequencies (beta and gamma). Conversely, in the post‐section, synchronized oscillations were more prominent in the higher frequencies (beta and gamma) than in the lower frequencies.
- Similar results were obtained when comparing section EZ with section LZ.
- In comparing section MZ and section LZ, the functional connectivity in the pre‐section was higher in the low frequencies than in the higher frequencies. However, in the post‐section, a higher degree of synchronized oscillation was recorded in the following order: delta > gamma > beta > alpha > theta.

For further details, refer to Table 1, which provides information on the number of functional connectivity with *r >* 0.7, 0.75, and 0.8. This table shows how the selection criteria of *r* affect the number of functional connectivity.

**TABLE 1:** Number of functional connectivity among three distinct sections (early Zentangle [EZ], mid Zentangle [MZ], and late Zentangle [LZ]) with different selection criteria.

*r*	Pre‐section vs. post‐section	Delta	Theta	Alpha	Beta	Gamma
*r* > 0.7	EZ vs. MZ	263, 16	147, 5	103, 8	72, 31	67, 21
	EZ vs. LZ	293, 18	147, 19	110, 16	96, 27	107, 29
	MZ vs. LZ	45, 73	47, 33	40, 39	28, 57	30, 62
*r* > 0.75	EZ vs. MZ	204, 2	116, 1	86, 3	91, 10	69, 17
	EZ vs. LZ	221, 5	122, 4	101, 13	113, 19	94, 22
	MZ vs. LZ	32, 18	17, 14	27, 22	38, 25	47, 27
*r* > 0.8	EZ vs. MZ	104, 1	77, 0	67, 0	51, 7	50, 6
	EZ vs. LZ	119, 2	86, 0	80, 4	70, 7	71, 7
	MZ vs. LZ	18, 4	13, 4	15, 6	25, 6	25, 7

**FIGURE 7:**
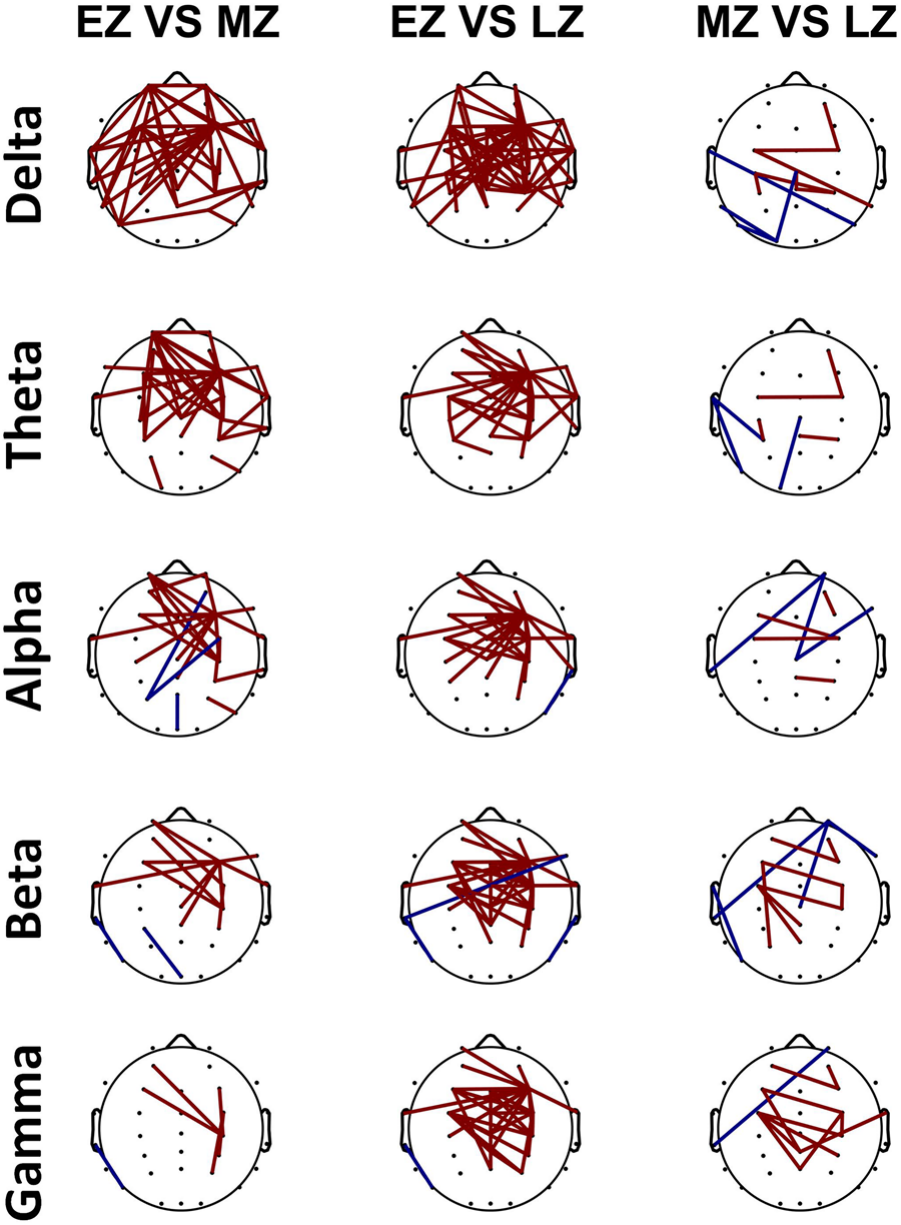
Functional connectivity among three distinct sections (early Zentangle [EZ], mid Zentangle [MZ], and late Zentangle [LZ]) in different frequency bands. The threshold value of coherence is *r* > 0.7, and Wilcoxon sign rank test is applied to paired of channels. Red indicates stronger interelectrode coherence in the pre‐section compared to the post‐section, whereas blue represents the reverse situation.

Additionally, the Wilcoxon sign rank test was applied to select only those pairs of channels that showed significant differences between post‐ and pre‐section. We plot these results in Figure 7 which also shows the same trend of greater functional connectivity in section EZ as compared to sections MZ and LZ. In the comparison of section EZ with MZ, the pre‐section shows a high number of functional connectivity in the low‐frequency band (delta, theta, and alpha) as compared to the higher‐frequency band, whereas the post‐section has no functional connectivity in the high‐frequency band (delta and theta). In section EZ versus LZ, the pre‐section shows strong functional connectivity in all frequency bands as compared to post‐section. Similarly, compared to section MZ with LZ, there is a strong, synchronized oscillation in the pre‐section compared to the position section in all frequency bands.

## 4 DISCUSSION

To the best of our knowledge, this study represents the first attempt to investigate the effects of a Zentangle intervention on participants’ cognitive focus, emotional well‐being, stress levels, neural activity patterns, and functional connectivity within distinct brain regions and frequency bands. The results derived from questionnaire data, PSD comparison, ERSP analysis, and functional connectivity analysis have provided valuable insights into the impact of the intervention on participants’ mental states and brain activity.

The findings from the questionnaire data analysis demonstrated statistically significant differences in participants’ levels of CR, ECR, and SAR across three distinct sections of the Zentangle experiment. Specifically, CR and ECR significantly improved from section EZ to sections MZ and LZ, whereas SAR decreased significantly from section EZ to section LZ. These results showed that the Zentangle intervention positively impacted participants’ cognitive focus and emotional well‐being. These findings are consistent with previous research, which supports the efficacy of Zentangle and mindfulness‐based art therapies in enhancing mental well‐being, reducing stress, and promoting self‐efficacy, particularly among healthcare professionals and older adults (Chan & Lo, 2024; Cheung et al., 2023; Chung et al., 2022; Hsu et al., 2021; Sit et al., 2022). These findings align with previous studies investigating different mindfulness methods for stress reduction (Bohlmeijer et al., 2010; Irving et al., 2009; Schell et al., 2019), emotional calmness promotion (Hill & Updegraff, 2012; Luberto et al., 2021), and depression (Desrosiers et al., 2013; Thompson et al., 2015) and anxiety management (Brown & Ryan, 2003; Cotton et al., 2020).

The comparison of PSD and ERSP analysis yielded consistent results, revealing distinct neural activity patterns across various brain regions and frequency bands, except for the delta band, where PSD did not show any significant difference. In the PSD analysis, significant differences emerged in different frequency bands between experimental sections, revealing variations in theta, alpha, beta, and gamma bands among frontal, central, parietal, and occipital loops across different sections. Meanwhile, ERSP analysis showed EEG power shifts across section EZ, with reductions in delta power and increases in theta power in sections MZ and LZ compared to section EZ. Significant power increases in alpha, beta, and gamma bands also highlighted changes in neural activity patterns. These results are consistent with previous research showing decreased delta band (Gao et al., 2016; Jaiswal et al., 2019; Ng et al., 2021) and increased theta (Ahani et al., 2014; Cahn & Polich, 2006; Chiesa & Serretti, 2010; Ivanovski & Malhi, 2007; Lomas et al., 2015), alpha (Ahani et al., 2014; Chiesa & Serretti, 2010; Gao et al., 2016; Ivanovski & Malhi, 2007; Lomas et al., 2015), beta (Ahani et al., 2014; Gao et al., 2016; Ivanovski & Malhi, 2007; Ng et al., 2021), and gamma (Ferrarelli et al., 2013) band power during mindfulness meditation.

Our results indicate significant reductions in delta power and increase in theta, alpha, beta, and gamma power, which are consistent with the neuropsychological effects associated with mindfulness meditation. Interestingly, these patterns closely resemble those observed in mindfulness meditation. For instance, following mindfulness meditation, individuals often exhibit low delta and high alpha frequencies associated with reduced anxiety levels, improved conflict control, and enhanced working memory (Jaiswal et al., 2019). Additionally, our finding of heightened theta activity aligns with observations in older adults and athletes, where stress and anxiety levels decrease after mindfulness meditation (Ahani et al., 2014; Nien et al., 2023). The co‐occurrence of increased theta and alpha wave activity in our study suggests a state of relaxed alertness, potentially promoting concentration and emotional calm, both conducive to positive mental health (Lomas et al., 2015; Nien et al., 2023). Furthermore, the increased theta power correlates with improved attention, diminished anxiety, sympathetic autonomic nervous system activity, and enhanced memory functions, whereas the increased alpha power aligns with reduced anxiety, heightened feelings of calmness, and positive affect (Jensen et al., 2014). Notably, an increase in beta activity is correlated with heightened alertness, whereas gamma activity is involved in processing meaningful stimuli and integrating sensory input into a coherent whole, thereby enhancing attention ability after mindfulness practice (Berkovich‐Ohana et al., 2012; Stern et al., 2001).

The results of the functional connectivity analysis showed that EZ exhibited a higher degree of synchronized oscillations and more functional connections across all frequency bands compared to MZ and LZ. This aligns with a previous study suggesting that mindfulness meditation can reduce functional connectivity between brain regions, as evidenced by reduced lagged coherence across various meditation practices and frequency ranges. This suggests a potential reduction in self‐process interaction, implying detachment and dissolution of ego boundaries during meditation (Lehmann et al., 2012). Another study found that mindfulness meditation, specifically breath counting, reduced intracortical lagged coherence but increased head‐surface conventional coherence, showing heightened bodily attention without cognitive reasoning, differing from experienced meditators’ reductions (Milz et al., 2014). Table 2 summarizes the study's key findings on the effects of Zentangle intervention on neural activity patterns and functional connectivity in the brain.

**TABLE 2:** Summary of the key findings of the study on the effects of Zentangle intervention on neural activity patterns and functional connectivity in the brain.

Finding	Brain region	Frequency band	Implications
Decreased delta power	Entire scalp	Delta band	Indicates a state of relaxed alertness and better conflict control conducive to positive mental health (Jaiswal et al., 2019)
Increased theta power	Frontal, central, parietal, and occipital loops	Theta band	Indicates improved attention, diminished anxiety, sympathetic autonomic nervous system activity, and enhanced memory functions (Ahani et al., 2014; Jensen et al., 2014; Lomas et al., 2015)
Increased alpha power	Frontal, central, parietal, and occipital loops	Alpha band	Indicates reduced anxiety, heightened feelings of calmness, and positive affect (Jaiswal et al., 2019; Jensen et al., 2014; Lomas et al., 2015)
Increased beta power	Frontal, central, parietal, and occipital loops	Beta band	Indicates heightened alertness (Stern et al., 2001)
Increased gamma power	Frontal, central, parietal, and occipital loops	Gamma band	Indicates involvement in the processing of meaningful stimuli and the integration of sensory input into a coherent whole (Stern et al., 2001)
Decreased functional connectivity	–	All frequency bands	Indicates a potential reduction in self‐process interaction, implying detachment and dissolution of ego boundaries during meditation (Lehmann et al., 2012)

## 5 LIMITATIONS

Although this study offers initial insights into Zentangle's potential effects, some limitations require consideration. First, relying solely on self‐reported measures for concentration, emotional state, and stress (using a 10‐point Likert scale) introduces potential biases, such as social desirability or recall errors. Additionally, these self‐reported measures may not fully capture the physiological and neurological changes occurring during the Zentangle intervention. Second, the absence of objective physiological measures, such as heart rate variability (HRV) or cortisol levels, limits our understanding of the intervention's impact on the body's stress response and emotional regulation. Finally, the study included only male participants, restricting the generalizability of the findings to the broader population.

## 6 RECOMMENDATIONS

Incorporating subjective and objective measures is recommended to address the limitations and enhance future research. Alongside self‐reported experiences, collecting physiological data on HRV and cortisol levels could provide valuable insights into the potential physiological changes induced by Zentangle. Employing a longitudinal study design would further strengthen the research by allowing investigators to examine the sustainability of Zentangle's benefits over time. Finally, increasing the sample size and ensuring participant diversity across gender, age, and other relevant characteristics would enhance the generalizability of the research and solidify Zentangle's potential as a tool for promoting well‐being.

## 7 CONCLUSION

This study represents a groundbreaking exploration into the impact of Zentangle intervention on various aspects, including cognitive focus, emotional well‐being, stress levels, neural activity patterns, and functional connectivity across distinct brain regions and frequency bands. In summary, the results suggest that Zentangle shows great promise as a mindfulness meditation practice for improving concentration and emotional calmness while simultaneously reducing stress levels. The observed changes in neural activity patterns, spanning delta, theta, alpha, beta, and gamma bands align closely with the neuropsychological effects commonly associated with mindfulness meditation, in line with prior research findings. Furthermore, the functional connectivity analysis reveals a decrease in self‐process interaction, indicating that Zentangle promotes a state of mindful detachment. This study provides valuable contributions to the growing body of knowledge on holistic approaches to mental well‐being, highlighting the potential of Zentangle as a tool for improving mental health.

## AUTHOR CONTRIBUTIONS

**Muhammad Usman**: Methodology; software; data curation; formal analysis; writing―original draft; writing―review and editing; conceptualization; investigation. **Tzzy‐Ping Jung**: Writing―review and editing; supervision; validation. **Ding‐Yun Hsin**: Methodology; writing―review and editing. **Chun‐Ling Lin**: Writing―review and editing; methodology; software; supervision; funding acquisition.

### PEER REVIEW

The peer review history for this article is available at https://publons.com/publon/10.1002/brb3.3628.

## Supporting information


**Table A** Summary of self‐reported questionnaire collected from 30 subjects before and during the Zentangle experiment (EZ, MZ, and LZ).

## Data Availability

The data that support the findings of this study are available on request from the corresponding author. The data are not publicly available due to privacy or ethical restrictions.
