# Supplementary material for: The effect of Zentangle on cognitive focus, emotional well‐being, and stress levels: A neural perspective
Source: Brain Behav. 2024 Aug 21;14(8):e3628. doi: 10.1002/brb3.3628 (PMC11338741; doi:10.1002/brb3.3628)
Supplement: Supplementary file 1 — Table A Summary of self‐reported questionnaire collected from 30 subjects before and during the Zentangle experiment (EZ, MZ, and LZ). [file BRB3-14-e3628-s001.docx]

Table A. Summary of self-reported questionnaire collected from 30 subjects before and during the Zentangle experiment (EZ, MZ, and LZ).

| Subject | Pre Zentangle | | | Increase CR | CR | | | Increase ECR | ECR | | | Decrease SAR | SAR | | |
| --- | --- | --- | --- | --- | --- | --- | --- | --- | --- | --- | --- | --- | --- | --- | --- |
|  | CR | ECR | SAR |  | EZ | MZ | LZ |  | EZ | MZ | LZ |  | EZ | MZ | LZ |
| 1 | 3 | 3 | 4 | N | 3 | 3 | 3 | Y | 6 | 6 | 6 | Y | 0 | 0 | 0 |
| 2 | 4 | 2 | 3 | Y | 6 | 7 | 9 | Y | 4 | 5 | 3 | Y | 0 | 1 | 0 |
| 3 | 4 | 4 | 0 | N | 4 | 4 | 4 | N | 4 | 4 | 4 | N | 0 | 0 | 0 |
| 4 | 5 | 5 | 0 | N | 5 | 5 | 5 | N | 5 | 5 | 5 | N | 0 | 0 | 0 |
| 5 | 4 | 4 | 3 | Y | 8 | 9 | 9 | Y | 8 | 9 | 9 | Y | 0 | 1 | 1 |
| 6 | 4 | 3 | 5 | Y | 7 | 7 | 8 | Y | 5 | 6 | 7 | Y | 3 | 2 | 0 |
| 7 | 4 | 4 | 2 | Y | 7 | 8 | 9 | Y | 7 | 9 | 9 | Y | 1 | 0 | 0 |
| 8 | 5 | 5 | 2 | N | 5 | 5 | 5 | N | 5 | 5 | 5 | N | 2 | 2 | 2 |
| 9 | 3 | 4 | 0 | Y | 6 | 7 | 8 | Y | 8 | 8 | 9 | N | 0 | 0 | 0 |
| 10 | 3 | 4 | 3 | Y | 6 | 7 | 8 | Y | 8 | 8 | 9 | Y | 0 | 1 | 1 |
| 11 | 3 | 3 | 5 | Y | 6 | 7 | 7 | Y | 6 | 7 | 6 | Y | 5 | 4 | 3 |
| 12 | 2 | 3 | 4 | Y | 2 | 7 | 5 | Y | 6 | 5 | 4 | Y | 3 | 3 | 2 |
| 13 | 5 | 5 | 4 | Y | 5 | 9 | 5 | Y | 8 | 9 | 7 | N | 4 | 4 | 4 |
| 14 | 3 | 3 | 4 | Y | 5 | 6 | 8 | Y | 4 | 5 | 6 | Y | 3 | 1 | 0 |
| 15 | 1 | 4 | 4 | Y | 3 | 5 | 2 | N | 4 | 4 | 4 | Y | 0 | 1 | 1 |
| 16 | 3 | 4 | 2 | Y | 5 | 7 | 8 | Y | 5 | 6 | 6 | Y | 1 | 0 | 0 |
| 17 | 3 | 3 | 3 | Y | 3 | 7 | 7 | Y | 3 | 6 | 5 | Y | 3 | 1 | 2 |
| 18 | 3 | 4 | 3 | Y | 6 | 7 | 6 | Y | 9 | 9 | 9 | Y | 1 | 0 | 0 |
| 19 | 3 | 4 | 4 | Y | 6 | 6 | 6 | Y | 8 | 8 | 8 | Y | 1 | 1 | 1 |
| 20 | 3 | 2 | 2 | N | 3 | 3 | 3 | N | 2 | 2 | 2 | N | 2 | 2 | 2 |
| 21 | 4 | 5 | 0 | Y | 8 | 9 | 9 | N | 5 | 5 | 5 | N | 0 | 0 | 0 |
| 22 | 3 | 5 | 0 | Y | 6 | 8 | 6 | Y | 9 | 10 | 9 | N | 0 | 0 | 0 |
| 23 | 3 | 3 | 0 | Y | 6 | 6 | 7 | Y | 6 | 6 | 8 | N | 0 | 0 | 0 |
| 24 | 3 | 3 | 1 | Y | 6 | 7 | 6 | Y | 3 | 7 | 8 | N | 1 | 1 | 1 |
| 25 | 3 | 4 | 1 | Y | 6 | 8 | 7 | Y | 8 | 7 | 8 | Y | 0 | 0 | 0 |
| 26 | 4 | 5 | 2 | Y | 7 | 8 | 6 | Y | 9 | 8 | 6 | Y | 1 | 1 | 0 |
| 27 | 3 | 4 | 3 | Y | 6 | 6 | 7 | Y | 7 | 7 | 7 | N | 3 | 3 | 3 |
| 28 | 3 | 4 | 0 | N | 3 | 3 | 3 | Y | 7 | 8 | 6 | N | 0 | 0 | 0 |
| 29 | 3 | 4 | 3 | Y | 5 | 7 | 8 | Y | 5 | 6 | 8 | Y | 1 | 1 | 0 |
| 30 | 3 | 3 | 3 | Y | 6 | 6 | 7 | Y | 5 | 7 | 8 | Y | 1 | 1 | 1 |

*Note*. Y and N represent yes and no respectively.

A self-reported questionnaire was collected from 30 subjects before Zentangle experiment and during the Zentangle experiment (EZ, MZ, and LZ).
